# 2-Methyl-3-(3-methyl­phen­yl)acrylic acid

**DOI:** 10.1107/S1600536808019545

**Published:** 2008-07-05

**Authors:** Niaz Muhammad, Saqib Ali, M. Nawaz Tahir, Zia-ur-Rehman

**Affiliations:** a Department of Chemistry, Quaid-i-Azam University, Islamabad 45320, Pakistan; b University of Sargodha, Department of Physics, Sagrodha, Pakistan

## Abstract

The crystal structure of the title compound, C_11_H_12_O_2_, consists of dimers which are formed due to inter­molecular O—H⋯O hydrogen bonding. The dimers are linked to each other by C—H⋯O hydrogen bonds, where C—H belongs to the benzene ring and the O atom is of a carbonyl group of an adjoining mol­ecule. There exist two inter­molecular C—H⋯O hydrogen bonds which form five-membered rings. There exist two π–π inter­actions between the benzene rings. The perpendicular distance in these inter­actions are 3.006 and 3.396 Å. There also exist C—H⋯π and C—O⋯π inter­actions.

## Related literature

For related literature, see: Bernstein *et al.* (1995 ▶); Liu *et al.* (1999 ▶); Muhammad *et al.* (2007 ▶); Natella *et al.* (1999 ▶); Niaz *et al.* (2008 ▶); Parez-Alvarez *et al.* (2001 ▶); Wiesner *et al.* (2001 ▶).
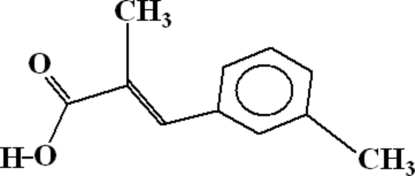


## Experimental

### 

#### Crystal data

C_11_H_12_O_2_
*M* *_r_* = 176.21
Monoclinic, 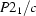

*a* = 7.4430 (9) Å
*b* = 13.4094 (16) Å
*c* = 10.2746 (12) Å
β = 110.745 (4)°
*V* = 959.0 (2) Å^3^
*Z* = 4
Mo *K*α radiation
μ = 0.08 mm^−1^
*T* = 296 (2) K
0.26 × 0.18 × 0.15 mm

#### Data collection

Bruker Kappa APEXII CCD diffractometer
Absorption correction: multi-scan (*SADABS*; Bruker, 2005 ▶) *T*_min_ = 0.980, *T*_max_ = 0.986
11342 measured reflections
2820 independent reflections
1075 reflections with *I* > 2σ(*I*)
*R*_int_ = 0.047

#### Refinement

*R*[*F* ^2^ > 2σ(*F* ^2^)] = 0.056
*wR*(*F* ^2^) = 0.185
*S* = 1.02
2820 reflections
120 parameters
H-atom parameters constrained
Δρ_max_ = 0.23 e Å^−3^
Δρ_min_ = −0.20 e Å^−3^

### 

Data collection: *APEX2* (Bruker, 2007 ▶); cell refinement: *APEX2*; data reduction: *SAINT* (Bruker, 2007 ▶); program(s) used to solve structure: *SHELXS97* (Sheldrick, 2008 ▶); program(s) used to refine structure: *SHELXL97* (Sheldrick, 2008 ▶); molecular graphics: *ORTEP-3 for Windows* (Farrugia, 1997 ▶) and *PLATON* (Spek, 2003 ▶); software used to prepare material for publication: *WinGX* publication routines (Farrugia, 1999 ▶) and *PLATON*.

## Supplementary Material

Crystal structure: contains datablocks global, I. DOI: 10.1107/S1600536808019545/at2580sup1.cif

Structure factors: contains datablocks I. DOI: 10.1107/S1600536808019545/at2580Isup2.hkl

Additional supplementary materials:  crystallographic information; 3D view; checkCIF report

## Figures and Tables

**Table 1:** Hydrogen-bond geometry (Å, °) *Cg* is the centroid of the C4–C9 benzene ring.

*D*—H⋯*A*	*D*—H	H⋯*A*	*D*⋯*A*	*D*—H⋯*A*
O1—H1⋯O2^i^	0.82	1.83	2.611 (3)	160
C3—H3⋯O1	0.93	2.27	2.703 (3)	108
C8—H8⋯O1^ii^	0.93	2.59	3.394 (3)	145
C10—H10*A*⋯O2	0.96	2.31	2.783 (3)	109
C10—H10*C*⋯*Cg*^iii^	0.96	2.75	3.610 (3)	149
C1—O2⋯*Cg*^iv^	1.25 (1)	3.57 (1)	3.895 (3)	95 (1)

Symmetry codes: (i) 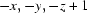; (ii) 
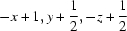
; (iii) 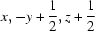; (iv) 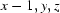.

**Fig. 1.**
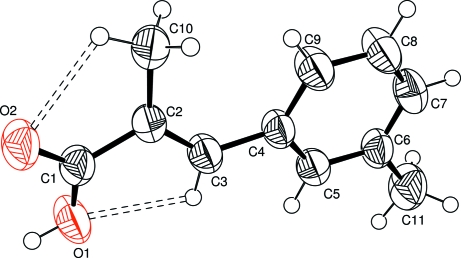
ORTEP drawing of the title compound, with the atom numbering scheme. The thermal ellipsoids are drawn at the 50% probability level. H-atoms are shown by small circles of arbitrary radii. The intramolecular H-bonds are shown by doted lines.

**Fig. 2.**
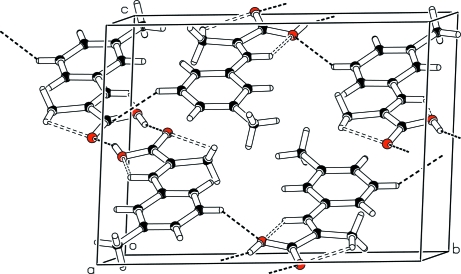
The packing figure (PLATON: Spek, 2003) which shows the dimeric nature of the compound and the interlinkages of the dimers.

## supplementary crystallographic information

### Comment

Cinnamic acids and their derivatives have been studied for their pharmacological properties, including hepatoprotactive (Parez-Alvarez *et al.*, 2001), antimalarial (Wiesner *et al.*, 2001), antioxident (Natella *et al.*, 1999) and antihyperglycemic activities (Liu *et al.*, 1999). In continuation of our efforts to synthesize various derivatives of cinamic acids (Niaz *et al.*, 2008) and their complexes, we herein report the structure of the title compound (I).

The crystal structure of 3-(4-bromophenyl)-2-methylacrylic acid (Muhammad *et al.*, 2007) has been reported. The title compound (I) have a similar environment about the carboxylate group but the attachement of methyl instead of Br-atom is at *meta*-position instead of *para*-position.

In the crystal structure of the title compound, the C—C bonds are in the range 1.462 (3)–1.500 (3) Å, and C═C have a value of 1.339 (3) Å. The resonant C—O bonds have values of 1.250 (3) and 1.271 (3) Å. In the asymmetric unit, there are two interamolecular H-bonds of C—H···O type (Table 2, Fig 1). Due to these H-bonds, two five membered rings (O1/C1/C2/C3/H3···O1) and (O2/C1/C2/C10/H10A···O2) are formed. The intermolecular (O1—H1···O2^i^ [symmetry code: i = -*x*, -*y*, -*z* + 1]) hydrogen bond forces the molecules into centrosymmetric dimers, forming a *R*_2_^2^(8) motif (Bernstein *et al.* 1995). These dimers are linked to each other by the second intermolecular H-bonding, C8—H8···O2^ii^ [symmetry code: ii = -*x* + 1, *y* + 1/2, -*z* + 1/2] as shown in Fig 2. There exist π–π interactions between the centroids (*Cg*) of benzene (C4—C9) rings of adjacent molecules. The *Cg*···*Cg*^iii^ [symmetry code: iii = *x*, -*y* + 1/2, *z* - 1/2] and Cg···Cg^iv^ [symmetry code: iv = *x*, -*y* + 1/2, *z* + 1/2] have a perpendicular distance of 3.006 and 3.396 Å, respectively. There exist also C10—H10C···*Cg*^iv^ interaction, with a distance of 3.610 (3) Å between C10 and *Cg*^iv^. Similarly another π-interaction is present between C1–O2 and *Cg*^v^ [symmetry code: v = *x* - 1, *y*, *z*] with a distance of 3.895 (3) Å between C1 and *Cg*^v^. The detail of these interactions is also included in Table 1.

### Experimental

Compound (I) was prepared according to our previously reported method (Muhammad *et al.*, 2007). A mixture of 3-methylbenzaldehyde (10 mmol, 1.18 ml), methylmalonic acid (2.36 g, 20 mmol) and piperidine (20 mmol, 1.98 ml) in pyridine (12.5 ml) solution was heated on a steam-bath for 24 h. The reaction mixture was cooled and added to a mixture of 25 ml of concentrated HCl and 50 g of ice. The precipitate formed in the acidified mixture was filtered off and washed with ice-cold water. The product was recrystallized from ethanol [yield; 90%, m.p. 321 K].

### Refinement

All H-atoms were positioned geometrically, with C—H = 0.93 and 0.96 Å for aromatic and methyl H-atoms, respectively, and O—H = 0.82 Å for hydroxyl O-atom, and constrained to ride on their parent atoms. The thermal parameters of methyl and hydroxyl H-atoms was taken 1.5 times while for all other H-atoms it was taken 1.2 times of the parent atoms.

### Crystal data

**Table:**

C_11_H_12_O_2_	*F*_000_ = 376
*M**_r_* = 176.21	*D*_x_ = 1.220 Mg m^−^^3^
Monoclinic, *P*2_1_/*c*	Mo *K*α radiation λ = 0.71073 Å
Hall symbol: -P 2ybc	Cell parameters from 2820 reflections
*a* = 7.4430 (9) Å	θ = 2.6–30.2º
*b* = 13.4094 (16) Å	µ = 0.08 mm^−^^1^
*c* = 10.2746 (12) Å	*T* = 296 (2) K
β = 110.745 (4)º	Prismatic, colourless
*V* = 959.0 (2) Å^3^	0.26 × 0.18 × 0.15 mm
*Z* = 4	

### Data collection

**Table:**

Bruker KAPPA APEXII CCD diffractometer	2820 independent reflections
Radiation source: fine-focus sealed tube	1075 reflections with *I* > 2σ(*I*)
Monochromator: graphite	*R*_int_ = 0.047
Detector resolution: 7.2 pixels mm^-1^	θ_max_ = 30.2º
*T* = 296(2) K	θ_min_ = 2.6º
ω scans	*h* = −10→8
Absorption correction: multi-scan(SADABS; Bruker, 2005)	*k* = −18→13
*T*_min_ = 0.980, *T*_max_ = 0.986	*l* = −10→14
11342 measured reflections	

### Refinement

**Table:**

Refinement on *F*^2^	Hydrogen site location: inferred from neighbouring sites
Least-squares matrix: full	H-atom parameters constrained
*R*[*F*^2^ > 2σ(*F*^2^)] = 0.056	*w* = 1/[σ^2^(*F*_o_^2^) + (0.0657*P*)^2^ + 0.191*P*] where *P* = (*F*_o_^2^ + 2*F*_c_^2^)/3
*wR*(*F*^2^) = 0.185	(Δ/σ)_max_ < 0.001
*S* = 1.02	Δρ_max_ = 0.24 e Å^−^^3^
2820 reflections	Δρ_min_ = −0.20 e Å^−^^3^
120 parameters	Extinction correction: empirical, Fc^*^=kFc[1+0.001xFc^2^λ^3^/sin(2θ)]^-1/4^
Primary atom site location: structure-invariant direct methods	Extinction coefficient: 0.006 (2)
Secondary atom site location: difference Fourier map	

### Special details

**Table:**

Geometry. Bond distances, angles *etc*. have been calculated using the rounded fractional coordinates. All su's are estimated from the variances of the (full) variance-covariance matrix. The cell e.s.d.'s are taken into account in the estimation of distances, angles and torsion angles
Refinement. Refinement of *F*^2^ against ALL reflections. The weighted *R*-factor *wR* and goodness of fit *S* are based on *F*^2^, conventional *R*-factors *R* are based on *F*, with *F* set to zero for negative *F*^2^. The threshold expression of *F*^2^ > σ(*F*^2^) is used only for calculating *R*-factors(gt) *etc*. and is not relevant to the choice of reflections for refinement. *R*-factors based on *F*^2^ are statistically about twice as large as those based on *F*, and *R*- factors based on ALL data will be even larger.

### Fractional atomic coordinates and isotropic or equivalent isotropic displacement parameters (Å^2^)

**Table:**

	*x*	*y*	*z*	*U*_iso_*/*U*_eq_	
O1	0.1630 (3)	−0.00578 (12)	0.4174 (2)	0.0795 (8)	
O2	0.0675 (2)	0.12222 (11)	0.51106 (18)	0.0699 (7)	
C1	0.1639 (3)	0.08682 (17)	0.4441 (2)	0.0515 (8)	
C2	0.2797 (3)	0.15393 (16)	0.3911 (2)	0.0484 (7)	
C3	0.4010 (3)	0.11136 (17)	0.3377 (2)	0.0533 (8)	
C4	0.5340 (3)	0.15354 (16)	0.2764 (2)	0.0497 (8)	
C5	0.6214 (3)	0.08770 (17)	0.2126 (2)	0.0534 (8)	
C6	0.7448 (3)	0.11877 (18)	0.1461 (2)	0.0553 (8)	
C7	0.7829 (4)	0.21882 (19)	0.1470 (3)	0.0637 (9)	
C8	0.7031 (4)	0.2856 (2)	0.2118 (3)	0.0699 (10)	
C9	0.5794 (4)	0.25412 (17)	0.2757 (3)	0.0646 (9)	
C10	0.2492 (3)	0.26372 (17)	0.4024 (3)	0.0658 (10)	
C11	0.8342 (4)	0.0464 (2)	0.0760 (3)	0.0788 (11)	
H1	0.10360	−0.03595	0.45872	0.0954*	
H3	0.40046	0.04203	0.33992	0.0639*	
H5	0.59610	0.01991	0.21458	0.0640*	
H7	0.86445	0.24181	0.10285	0.0764*	
H8	0.73305	0.35299	0.21244	0.0838*	
H9	0.52556	0.30031	0.31872	0.0774*	
H10A	0.15463	0.27413	0.44482	0.0988*	
H10B	0.20537	0.29296	0.31123	0.0988*	
H10C	0.36802	0.29433	0.45849	0.0988*	
H11A	0.79146	−0.01992	0.08522	0.1182*	
H11B	0.97157	0.04960	0.11877	0.1182*	
H11C	0.79701	0.06301	−0.02083	0.1182*	

### Atomic displacement parameters (Å^2^)

**Table:**

	*U*^11^	*U*^22^	*U*^33^	*U*^12^	*U*^13^	*U*^23^
O1	0.1052 (15)	0.0487 (10)	0.1240 (17)	−0.0062 (9)	0.0893 (13)	−0.0004 (10)
O2	0.0786 (12)	0.0602 (11)	0.0958 (13)	−0.0042 (8)	0.0617 (11)	−0.0100 (9)
C1	0.0537 (13)	0.0479 (13)	0.0629 (15)	0.0003 (10)	0.0330 (12)	−0.0028 (11)
C2	0.0460 (12)	0.0467 (12)	0.0570 (14)	−0.0032 (10)	0.0239 (11)	0.0020 (11)
C3	0.0583 (14)	0.0481 (12)	0.0634 (15)	−0.0046 (11)	0.0339 (12)	0.0003 (12)
C4	0.0510 (13)	0.0508 (12)	0.0549 (14)	−0.0052 (10)	0.0282 (12)	0.0009 (11)
C5	0.0525 (14)	0.0513 (13)	0.0611 (15)	−0.0035 (10)	0.0261 (12)	0.0021 (11)
C6	0.0490 (13)	0.0649 (16)	0.0589 (15)	−0.0034 (11)	0.0278 (12)	0.0047 (12)
C7	0.0631 (15)	0.0742 (17)	0.0646 (16)	−0.0139 (13)	0.0358 (14)	0.0053 (13)
C8	0.0812 (19)	0.0581 (15)	0.0849 (19)	−0.0194 (13)	0.0475 (17)	−0.0021 (13)
C9	0.0767 (17)	0.0525 (14)	0.0804 (18)	−0.0098 (12)	0.0475 (15)	−0.0045 (13)
C10	0.0574 (15)	0.0538 (15)	0.097 (2)	−0.0010 (11)	0.0408 (15)	−0.0017 (14)
C11	0.0757 (18)	0.088 (2)	0.091 (2)	0.0014 (15)	0.0521 (17)	−0.0051 (16)

### Geometric parameters (Å, °)

**Table:**

O1—C1	1.271 (3)	C8—C9	1.373 (4)
O2—C1	1.250 (3)	C3—H3	0.9300
O1—H1	0.8200	C5—H5	0.9300
C1—C2	1.477 (3)	C7—H7	0.9300
C2—C10	1.500 (3)	C8—H8	0.9300
C2—C3	1.339 (3)	C9—H9	0.9300
C3—C4	1.462 (3)	C10—H10A	0.9600
C4—C9	1.391 (3)	C10—H10B	0.9600
C4—C5	1.391 (3)	C10—H10C	0.9600
C5—C6	1.389 (3)	C11—H11A	0.9600
C6—C7	1.371 (4)	C11—H11B	0.9600
C6—C11	1.498 (4)	C11—H11C	0.9600
C7—C8	1.370 (4)		
			
C1—O1—H1	109.00	C4—C5—H5	119.00
O1—C1—O2	122.0 (2)	C6—C5—H5	119.00
O1—C1—C2	118.3 (2)	C6—C7—H7	119.00
O2—C1—C2	119.7 (2)	C8—C7—H7	119.00
C1—C2—C10	116.4 (2)	C7—C8—H8	120.00
C3—C2—C10	126.3 (2)	C9—C8—H8	120.00
C1—C2—C3	117.2 (2)	C4—C9—H9	120.00
C2—C3—C4	132.0 (2)	C8—C9—H9	120.00
C3—C4—C9	125.5 (2)	C2—C10—H10A	109.00
C5—C4—C9	117.3 (2)	C2—C10—H10B	109.00
C3—C4—C5	117.3 (2)	C2—C10—H10C	109.00
C4—C5—C6	122.9 (2)	H10A—C10—H10B	109.00
C5—C6—C11	121.8 (2)	H10A—C10—H10C	109.00
C7—C6—C11	120.8 (2)	H10B—C10—H10C	109.00
C5—C6—C7	117.5 (2)	C6—C11—H11A	109.00
C6—C7—C8	121.2 (3)	C6—C11—H11B	109.00
C7—C8—C9	120.7 (2)	C6—C11—H11C	110.00
C4—C9—C8	120.4 (2)	H11A—C11—H11B	109.00
C2—C3—H3	114.00	H11A—C11—H11C	109.00
C4—C3—H3	114.00	H11B—C11—H11C	109.00
			
O1—C1—C2—C3	−10.6 (3)	C9—C4—C5—C6	−2.0 (3)
O1—C1—C2—C10	169.3 (2)	C3—C4—C9—C8	−178.4 (2)
O2—C1—C2—C3	170.7 (2)	C5—C4—C9—C8	1.2 (4)
O2—C1—C2—C10	−9.4 (3)	C4—C5—C6—C7	1.3 (3)
C1—C2—C3—C4	179.6 (2)	C4—C5—C6—C11	−178.9 (2)
C10—C2—C3—C4	−0.3 (4)	C5—C6—C7—C8	0.4 (4)
C2—C3—C4—C5	−171.3 (2)	C11—C6—C7—C8	−179.5 (3)
C2—C3—C4—C9	8.3 (4)	C6—C7—C8—C9	−1.2 (4)
C3—C4—C5—C6	177.54 (19)	C7—C8—C9—C4	0.4 (4)

### Hydrogen-bond geometry (Å, °)

**Table:**

*D*—H···*A*	*D*—H	H···*A*	*D*···*A*	*D*—H···*A*
O1—H1···O2^i^	0.82	1.83	2.611 (3)	160
C3—H3···O1	0.93	2.27	2.703 (3)	108
C8—H8···O1^ii^	0.93	2.59	3.394 (3)	145
C10—H10A···O2	0.96	2.31	2.783 (3)	109
C10—H10C···Cg^iii^	0.9600	2.75	3.610 (3)	149.00
C1—O2···Cg^iv^	1.250 (3)	3.574 (2)	3.895 (3)	95.38 (13)

Symmetry codes: (i) −*x*, −*y*, −*z*+1; (ii) −*x*+1, *y*+1/2, −*z*+1/2; (iii) *x*, −*y*+1/2, *z*+1/2; (iv) *x*−1, *y*, *z*.
